# Global research trends on the links between glymphatic system and cognition: A bibliometric analysis (2009–2023)

**DOI:** 10.1097/MD.0000000000042166

**Published:** 2025-06-27

**Authors:** Xiaoqi Ying, Jingyang Xu, Qintao Yu, Xinru Wang, Songsen Lan, Liwan Hu, Ying Zhang, Dexiong Han

**Affiliations:** a The Third Clinical Medical College, Zhejiang Chinese Medical University, Hangzhou, China.

**Keywords:** bibliometrics, cognitive, glymphatic system, perivascular spaces, visualization

## Abstract

Since an increasing number of studies on glymphatic system (GS) have been published, this study aimed to evaluate the research trends, hotspots, and frontiers of GS in cognition. Publications from the Web of Science Core Collection database from January 1, 2009 to December 31, 2023 were screened. The analysis of annual publications, countries/regions, institutions, journals, authors, co-cited journals, co-cited authors, and keywords was conducted using CiteSpace and VOSviewer, the hotspots and major findings were summarized. In addition, ArcGIS software was used to display the number of publications by countries/regions. Six hundred twenty-five publications were included. Overall, the number of publications has been increasing steadily, these were published in 59 countries/regions and 149 institutions. Of these, American institutions had the highest number, and their international influence also ranked first. The journal “Neurology” published the most publications and was considered the most co-cited journal. The article entitled “The glymphatic pathway in neurological disorders” in Lancet neurology had the most citations. The keywords with the highest number of occurrences were “Alzheimer disease” and “small vessel disease,” which were regarded as research hotspots. Among 19 emergent terms, “Virchow robin spaces,” “vascular dementia,” and “autosomal dominant arteriopathy” were the first, and “white matter lesions” were the strongest. This bibliometric analysis reveals the rapid growth and evolving landscape of research on the GS in cognitive function. However, there remain significant knowledge gaps and methodological challenges that must be addressed to fully elucidate the mechanisms and implications of GS in cognitive impairment and neurodegeneration.

## 1. Introduction

Perivascular spaces, frequently referred to as Virchow–Robin spaces, were essential for maintaining brain health.^[1]^ Recently, a study argued that perivascular space dysfunction was closely associated with cognitive dysfunction.^[2]^ In addition, a research^[3]^ found in mice that a space formed between perivascular astrocytic endfeet and paravascular spaces has the function of regulating the transport of interstitial metabolic wastes, which further improved the clearance mechanism of the central nervous system and named it the glymphatic system (GS). The GS facilitated the flow of cerebrospinal fluid (CSF) into the brain along the perivascular spaces of the arteries and then into the interstitium facilitated by aquaporin 4 (AQP4) water channels.^[3,4]^ The pathway then directed the flow into the perivascular and perineural spaces of the veins, ultimately clearing solutes from the neurofibrillary network into the meninges and cervical lymphatic drains.^[3,5]^ Solutes included amyloid-beta (Aβ), tau,^[6]^ alpha-synuclein,^[7]^ and lactate.^[8]^ The bulk flow of CSF–interstitial fluid into the perivascular spaces delivers glucose^[9]^ and transports lipids, signaling molecules^[10]^ and apolipoprotein E^[11]^ for energy metabolism throughout the brain. Some studies have found that CSF flow patterns in the human brain are similar to those of the GS in rodents^[12,13]^ suggesting that the GS also exists in humans. Protein aggregates were a common feature in patients with Alzheimer disease (AD), Parkinson disease, and other neurodegenerative diseases.^[6,14,15]^ This suggested that reduced brain clearance could be a shared phenomenon in neurodegeneration. Cerebral small vessel disease was a common cerebrovascular pathology, in which neuroimaging may reveal recent small subcortical infarcts, lacunes, white-matter hyperintensities, perivascular spaces, microbleeds, and cerebral atrophy.^[16,17]^ The most common causes of cognitive impairment were neurodegenerative and cerebrovascular pathology^[18]^ and neuroimaging features can also provide evidence.^[19,20]^ And the dysfunction of the GS can lead to the accumulation of brain waste, thus contributing to the progress of neurodegenerative and cerebrovascular pathology, leading to cognitive impairment.

However, to date, no research has summarized and analyzed the literature on the GS and cognition using bibliometric methods. Thus, this is the first study used the bibliometric method to summarize and visually analyze the articles on the GS in cognition published in the past 15 years. Findings of this study can provide an overall understanding and insights for future research.

## 2. Materials and methods

### 2.1. Data collection

We took full consideration of the possible alternative descriptions of GS, using TS=(“glymphatic system” OR “Glymphatic Pathway” OR “Meningeal Lymphatic Vessel” OR Brain Perivascular Space OR Virchow–Robin Space) AND “cognitive”) as search strategy. The search period ranges from January 1, 2009 to December 31, 2023 in the Web of Science Core Collection database. Publications types were limited to articles and reviews. The research index should include the GS or the space around blood vessels and cognition.

### 2.2. Analytical tool

**Figure 1.**
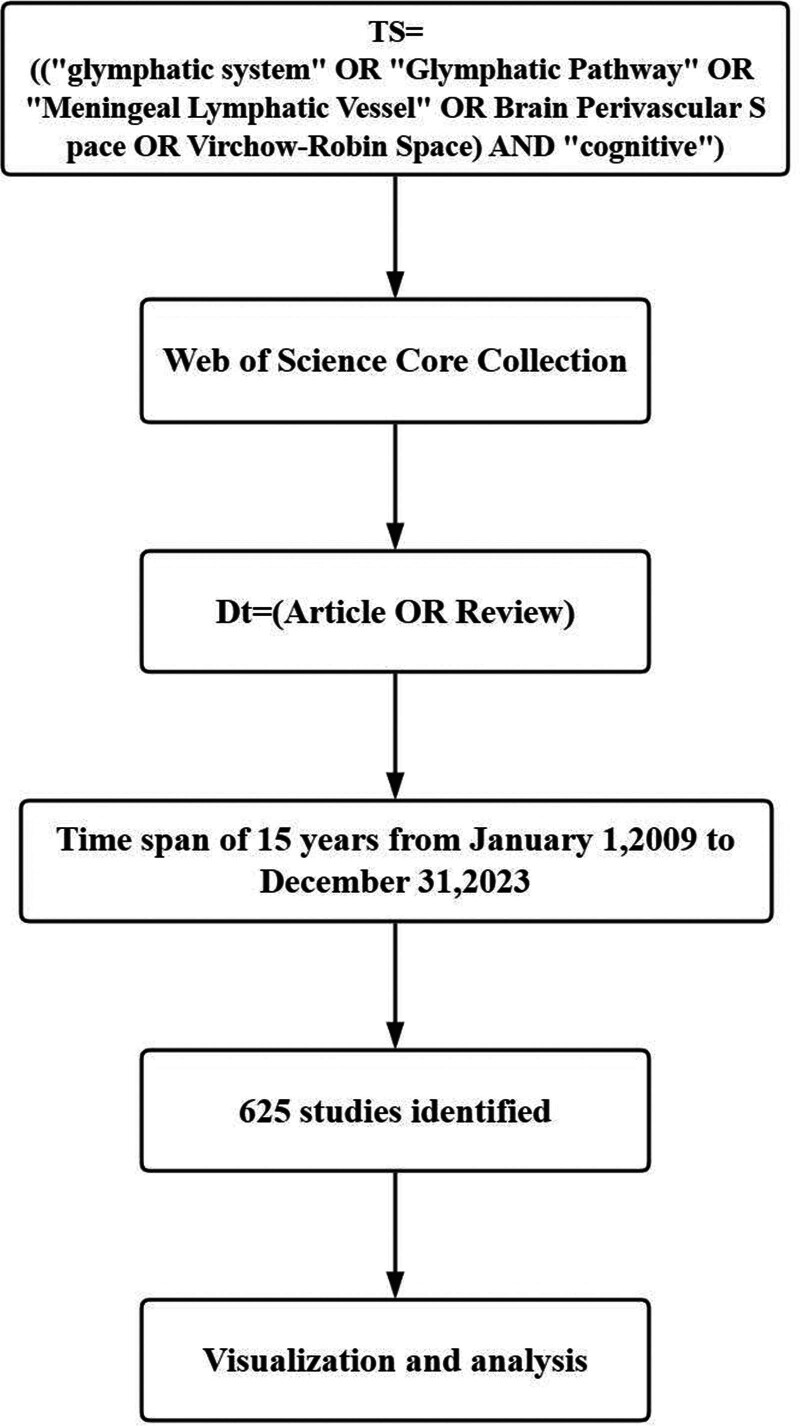
Flow chart of publication screening.

The full record and cited references of the publications were downloaded, and imported into Microsoft Excel (version 2019) to manage the data. Two researchers were involved in literature review and data selection. Visual analysis was performed using CiteSpace (version 6.2.R4), VOSviewer (version 1.6.20), and ArcGIS pro (version 3.0.2). CiteSpace and VOSviewer were used to visualize and analyze the data, providing an intuitive understanding of the essential data and the research hotspots of the GS in cognition and predicted its future development.^[21,22]^ Geographic information system application was used to display the number of publications by countries/regions (Fig. 1).

## 3. Results

### 3.1. Publication trend analysis

**Figure 2.**
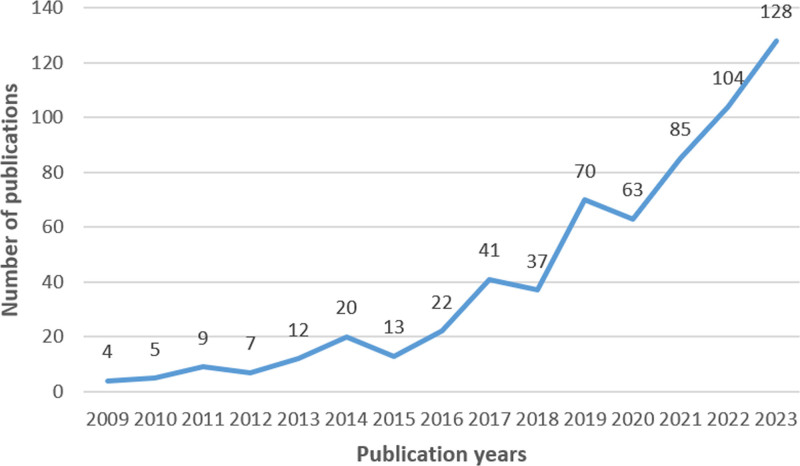
Publication trend.

A total of 625 publications met the inclusion criteria, consisting of 455 articles and 170 reviews. According to Figure 2, there was an overall upward trend in the number of annual publications from 2009 to 2023. The lowest number of publications was in 2009, with 4 publications, while the highest number of publications was in 2023, with 128 publications. There was a highlight that after the concept of GS was put forward in 2012, the number of publications has gradually increased, especially from 2017 to 2023, showing the increasing attention from researchers in recent years. However, reviews accounted for a significant proportion of publications (27.2%), and more innovative studies will be needed in the future.

### 3.2. Countries or regions analysis

**Figure 3.**
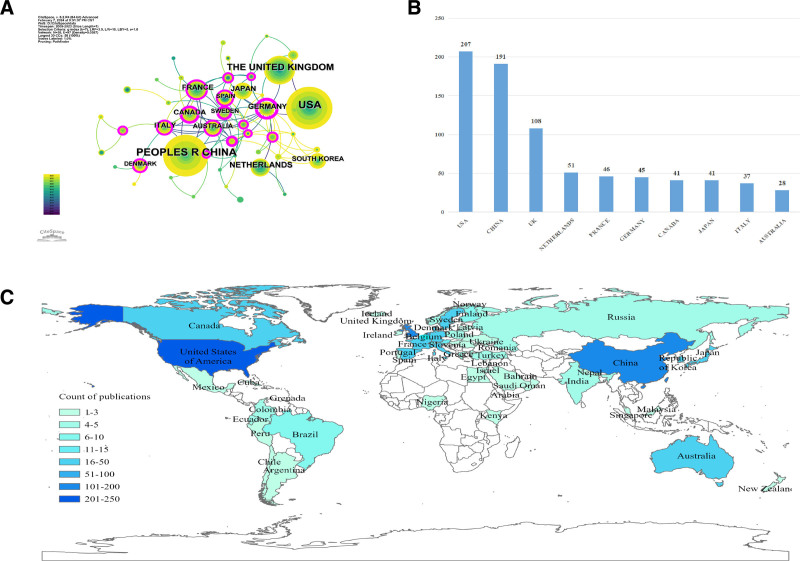
Countries or regions analysis. (A) Countries/regions network analysis; (B) number of publications in the top 10 countries/regions; (C) geographical distribution map of national/regional publications.

Research on GS and cognitive function was conducted in 75 countries or regions from 2009 to 2023. The countries or regions’ collaboration network was constructed using CiteSpace (Fig. 3A). Each node represented a country. Nodes with purple rings represented high centrality (centrality ≥ 0.1), indicating that they were influential and their publications were highly cited within a short period.^[21]^ The USA ranked first with 207 publications, and China and the United Kingdom followed with 191 and 108 publications, respectively. The top 10 countries or regions in the number of publications are displayed in Figure 3B. The geographic distribution of publications produced by countries or regions is shown in Figure 3C.

### 3.3. Institutions analysis

**Figure 4.**
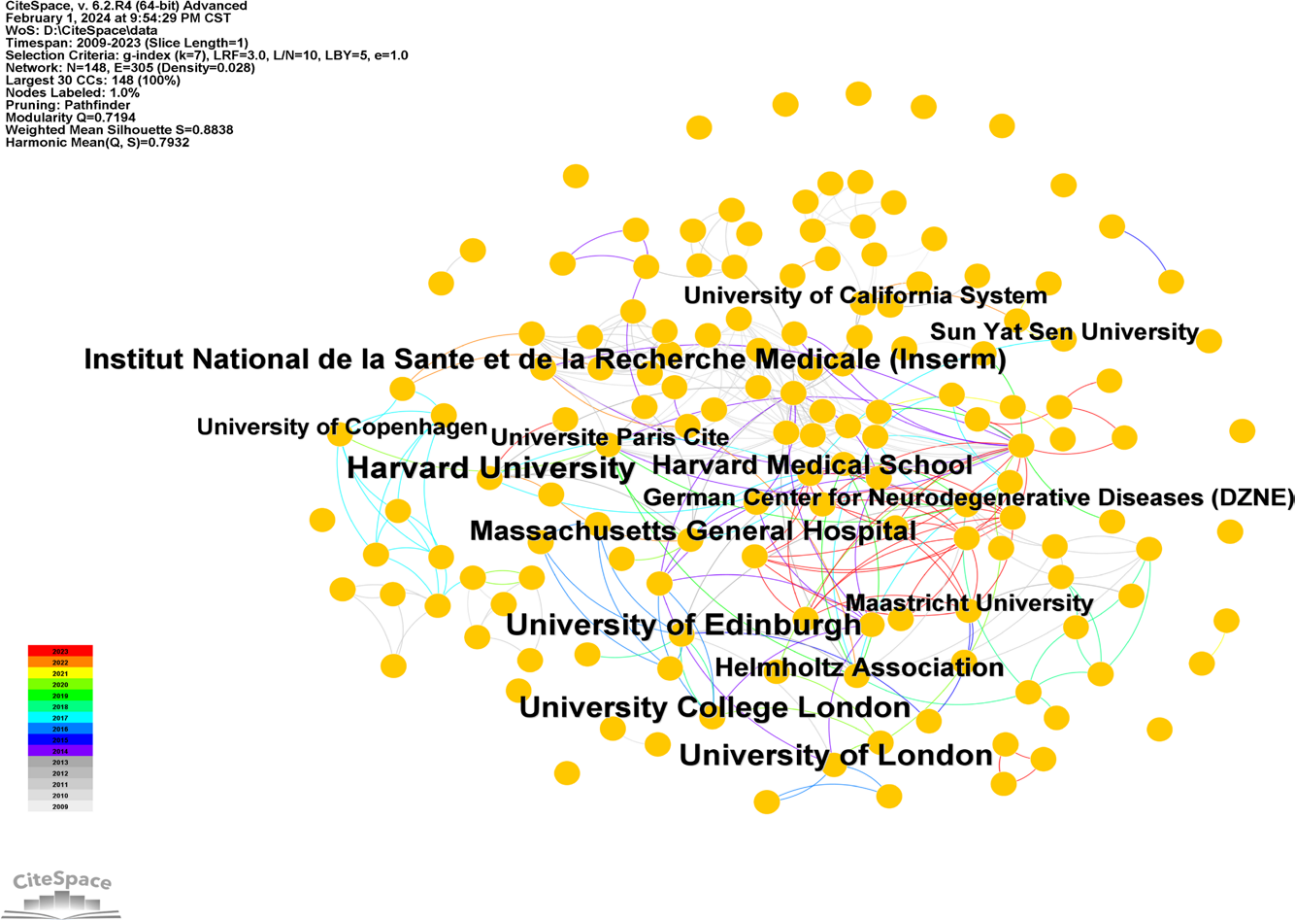
Institutions collaboration network.

Research on GS in cognition was conducted in 149 institutions from 2009 to 2023. The institution’s collaboration network was constructed using CiteSpace (Fig. 4). The same meaning keywords were merged: (1) Harvard University and Harvard Medical School; (2) University of London and University College London. The top 10 institutions with the highest number of publications are shown in Table 1. The majority of those with the most publications are organizations from the UK, followed by the USA. While China has a high volume of publications, there was no close cooperation between the organizations. Among these, Harvard University (37 publications), the University of Edinburgh (35 publications), and the University of London (34 publications) were the top 3 institutions in terms of the number of published studies.

**Table 1:** Top 10 institutions.

Count	Centrality	Year	Institutions
37	0.06	2013	Harvard University
35	0.15	2011	University of Edinburgh
34	0.06	2011	University of London
30	0.01	2011	University College London
27	0.02	2010	Institut National de la Sante et de la Recherche Medicale (Inserm)
26	0.19	2013	Massachusetts General Hospital
22	0.09	2016	Harvard Medical School
19	0.24	2011	Helmholtz Association
15	0.06	2013	Maastricht University
15	0.02	2017	Sun Yat Sen University

### 3.4. Journals and co-cited journals analysis

**Figure 5.**
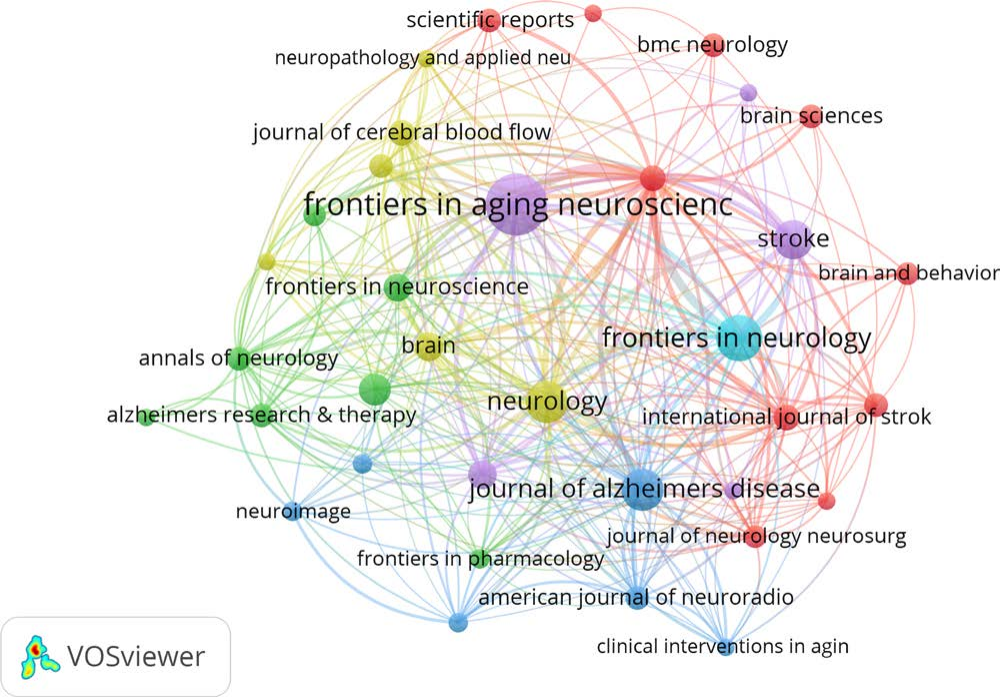
Cited journal analysis.

**Figure 6.**
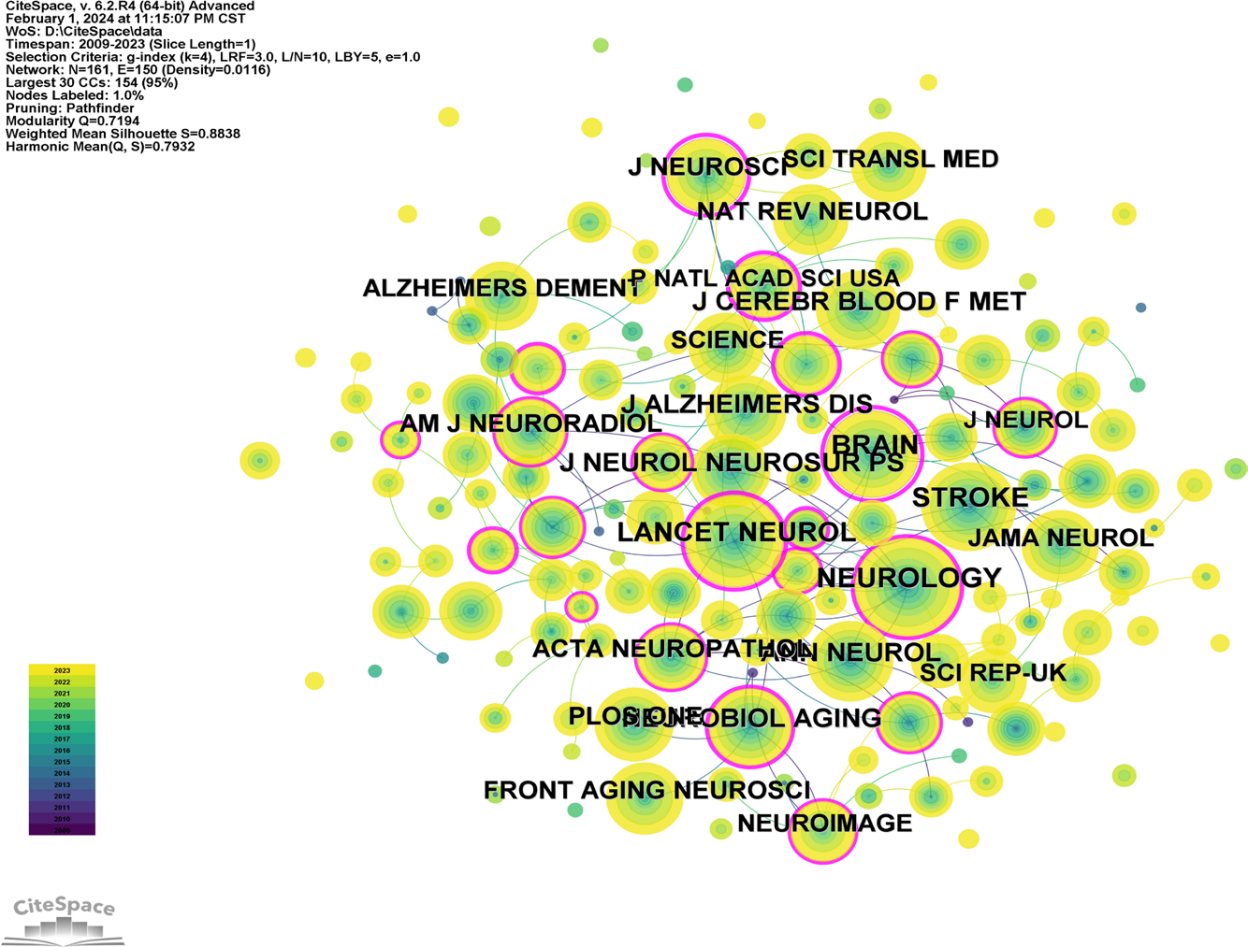
Co-cited journal analysis.

VOSviewer was used to analyze cited journals (Fig. 5) and Citespace was used to analyze co-cited journals (Fig. 6). Between 2009 and 2023, research on GS and cognition was published in 253 academic journals. Among them, “Frontiers in aging neuroscience” ranked first with 41 publications followed by frontiers in neurology with 24 publications. A co-citation can reveal influential journals in a particular field by measuring the frequency with which 2 journals are cited together in the same publication.^[23]^ Among co-cited journals, Neurology (507), Lancet Neurology (435), and Stroke (433) were the top 3 co-cited journals, indicating their high academic prestige in the field of GS in cognition (Table 2).

**Table 2:** Top 10 journals and co-cited journals.

Journal	Publication count	Journal	Cited Count
Frontiers in aging neuroscience	41	Neurology	507
Frontiers in neurology	24	Lancet neurology	445
Journal of Alzheimers disease	20	Stroke	433
Neurology	20	Brain	393
Stroke	17	Annals of neurology	359
International journal of molecular sciences	12	Journal of alzheimers disease	327
Brain	10	Neurobiology of Aging	327
Neurobiology of aging	10	Journal of neurology neurosurgery and psychiatry	325
Frontiers in neuroscience	9	Journal of cerebral blood flow and metabolism	308
International journal of stroke	8	Plos one	285

### 3.5. Authors and co-cited authors analysis

**Figure 7.**
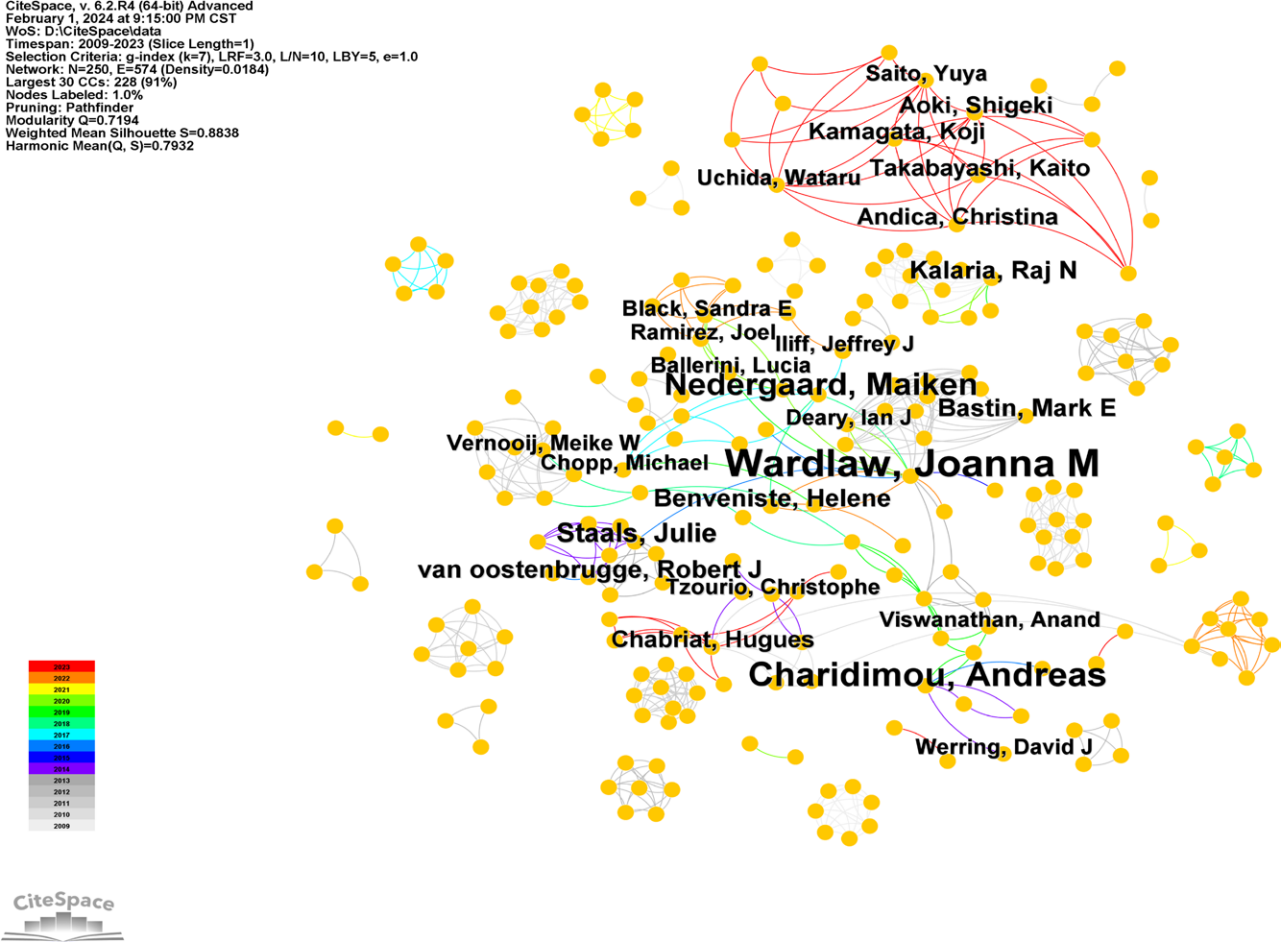
Authors collaboration network.

**Figure 8.**
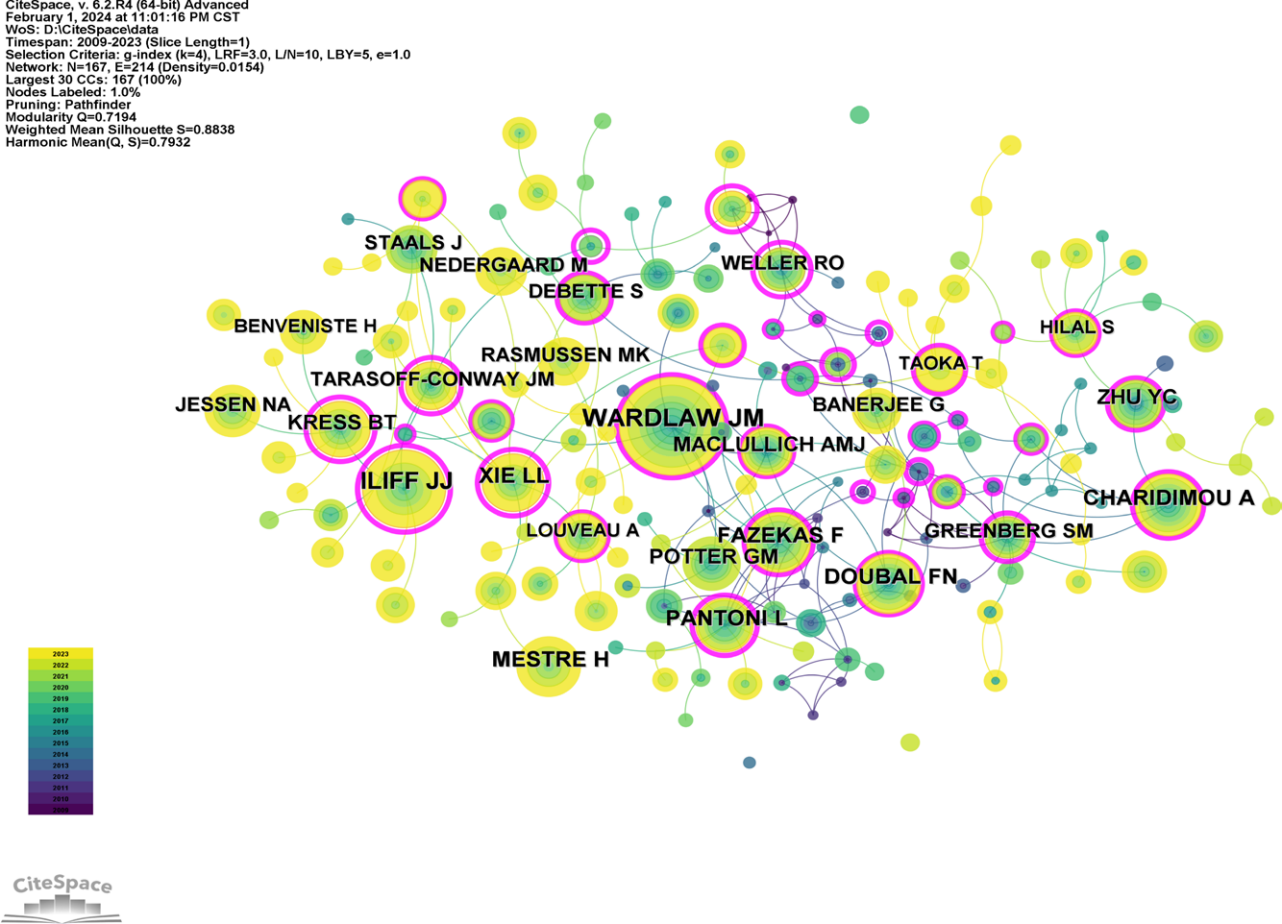
Co-cited author’s network.

Citespace was used to analyze the collaboration of authors (Fig. 7) and co-cited authors (Fig. 8). The count, centrality, and year of authors and co-cited authors are listed in Table 3. More collaborative communication between authors was evident in the analysis. The authors with the most publications were Wardlaw, Joanna M (n = 25), followed by Charidimou, Andreas (n = 16) and Nedergaard, Maiken (n = 13). Wardlaw, Joanna M was also the most frequently co-cited author (306), showing the research strength and hot topics. Ilff JJ (210) and Pantoni L (146) also had highly cited publications.

**Table 3:** Top 10 authors and co-cited authors.

Rank	Count	Authors	Rank	Count	Cited authors
1	25	Wardlaw JM	1	306	Wardlaw JM
2	16	Charidimou A	2	210	Iliff JJ
3	13	Nedergaard M	3	146	Pantoni L
4	8	Staals J	4	139	Doubal FN
5	7	Kalaria RN	5	136	Fazekas F
6	7	Bastin ME	6	133	Charidimou A
7	7	van Oostenbrugge RJ	7	124	Xie LL
8	7	Benveniste H	8	117	Mestre H
9	6	Andica C	9	108	Kress BT
10	6	Aoki S	10	101	Potter GM

### 3.6. Most cited articles analysis

The top 10 cited articles are shown in Table 4. Nine out of 10 were published in journals classified as Q1 and with IFs >10. Three of the top 10 cited articles were from Wardlaw JM and 2 were from Mestre H. The article entitled “The glymphatic pathway in neurological disorders” published in Lancet neurology had the most citations.

**Table 4:** Top 10 cited articles.

Citation	Centrality	Year	Title	First author	Journal
72	0.06	2018	The glymphatic pathway in neurological disorders	Rasmussen MK	Lancet neurology
61	0.07	2020	Perivascular spaces in the brain: anatomy, physiology and pathology	Wardlaw JM	Nature Reviews Neurology
54	0.19	2013	Neuroimaging standards for research into small vessel disease and its contribution to aging and neurodegeneration	Wardlaw JM	Lancet neurology
49	0.07	2020	Glymphatic failure as a final common pathway to dementia	Nedergaard M	Science
43	0.18	2019	Small vessel disease: mechanisms and clinical implications	Wardlaw JM	Lancet neurology
43	0.11	2018	Flow of cerebrospinal fluid is driven by arterial pulsations and is reduced in hypertension	Mestre H	Nature Communications
42	0.01	2018	Aquaporin-4-dependent glymphatic solute transport in the rodent brain	Mestre H	Elife
41	0.07	2018	Functional aspects of meningeal lymphatics in aging and Alzheimer disease	Da Mesquita S	Nature
39	0.03	2020	Impaired glymphatic function and clearance of tau in an Alzheimer disease model	Harrison Ian F	Brain
38	0.06	2017	Age and the fuzzy edges of embolic stroke of undetermined source: Implications for trials	Charidimou A	Neurology

### 3.7. Keyword co-occurrence, clustering analysis

**Figure 9.**
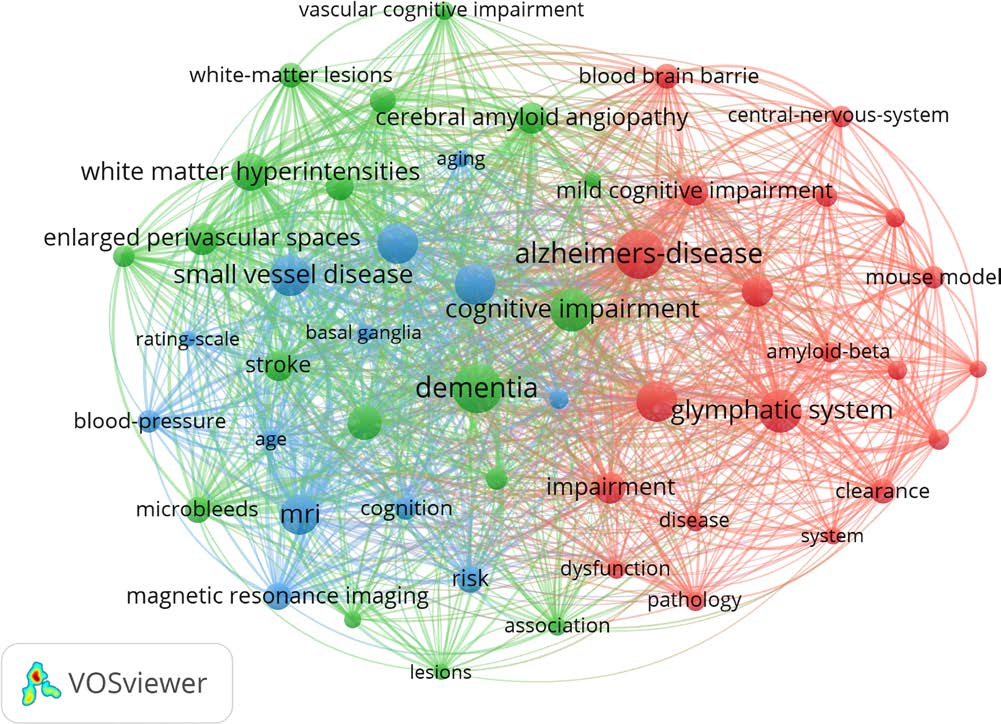
Keywords co-occurrence density map.

Keywords not only reflect the focus in a particular research area but also the heat and direction of research in a specific field. Keywords were summarized from the data in the Web of Science Core Collection and imported into CiteSpace. In our research, a total of 135 keywords were extracted and the total cumulative frequency of keywords amounted to 3219. Top 20 keywords as shown in Table 5. Vosviwer was uesd to analysis keywords hotspots (Fig. 9). Up to now research has focused more on the link between the GS and cognitive impairment, including “alzheimers disease” and “dementia,” and “small vessel disease” and “mri” are also hotspots. These different research hotspots contribute to optimizing and enhance the development of GS in cognition.

**Table 5:** Top 20 keywords.

Count	Key words	Centrality
203	Alzheimers disease	0.47
173	Small vessel disease	0.41
135	Cognitive impairment	0.05
132	Glymphatic system	0.03
126	Perivascular spaces	0.25
123	Dementia	0.22
118	Virchow–Robin spaces	0.33
100	Brain	0.05
93	MRI	0.2
93	White matter hyperintensity	0.27
92	Cerebral small vessel disease	0.03
78	Enlarged perivascular spaces	0.67
74	Impairment	0
72	Cerebral amyloid angiopathy	0.13
65	Risk factors	0
59	Mild cognitive impairment	0.31
56	Risk	0.06
53	Blood–brain barrier	0.2
52	White matter lesions	0.25
51	Magnetic resonance imaging	0.12

**Figure 10.**
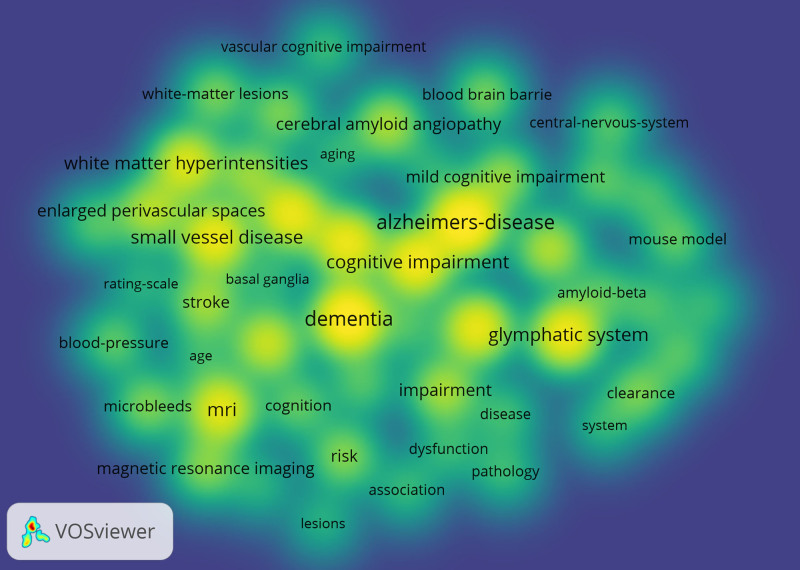
Keywords clustering network.

VOSviewer was used to perform clustering network analysis on keywords (Fig. 10). The same meaning keywords were merged: (1) AQP4, AQP-4, aquaporin-4 and aquaporin 4; (2) Alzheimers’-disease, Alzheimers-disease, Alzheimer-disease, and Alzheimer disease; (3) blood–brain barrier, blood–brain-barrier, and blood brain barrier; (4) white-matter hyperintensities and white matter hyperintensities. A total of 52 keywords were extracted, by defining keyword occurrences bigger than or equal to 25 as the extraction threshold and combining keywords with the same meaning. There were 3 main clusters: The red area is cluster 1, which contained 20 keywords with core cluster terms such as Alzheimers-disease, amyloid-beta, aquaporin 4, and blood–brain barrier. The clustering themes were vascular impairment on Alzheimers-disease. The green area is cluster 2, containing 18 keywords with core cluster terms such as association, microbleeds, and dementia. The clustering themes were cerebral small vessel disease on cognitive impairment such as dementia. The blue area is cluster 3, containing 13 keywords with core cluster terms such as age, MRI and small vessel diseases. The clustering themes were the impact of risk factors such as age on small vessel diseases and MRI.

### 3.8. Keywords timeline and emergence analysis

**Figure 11.**
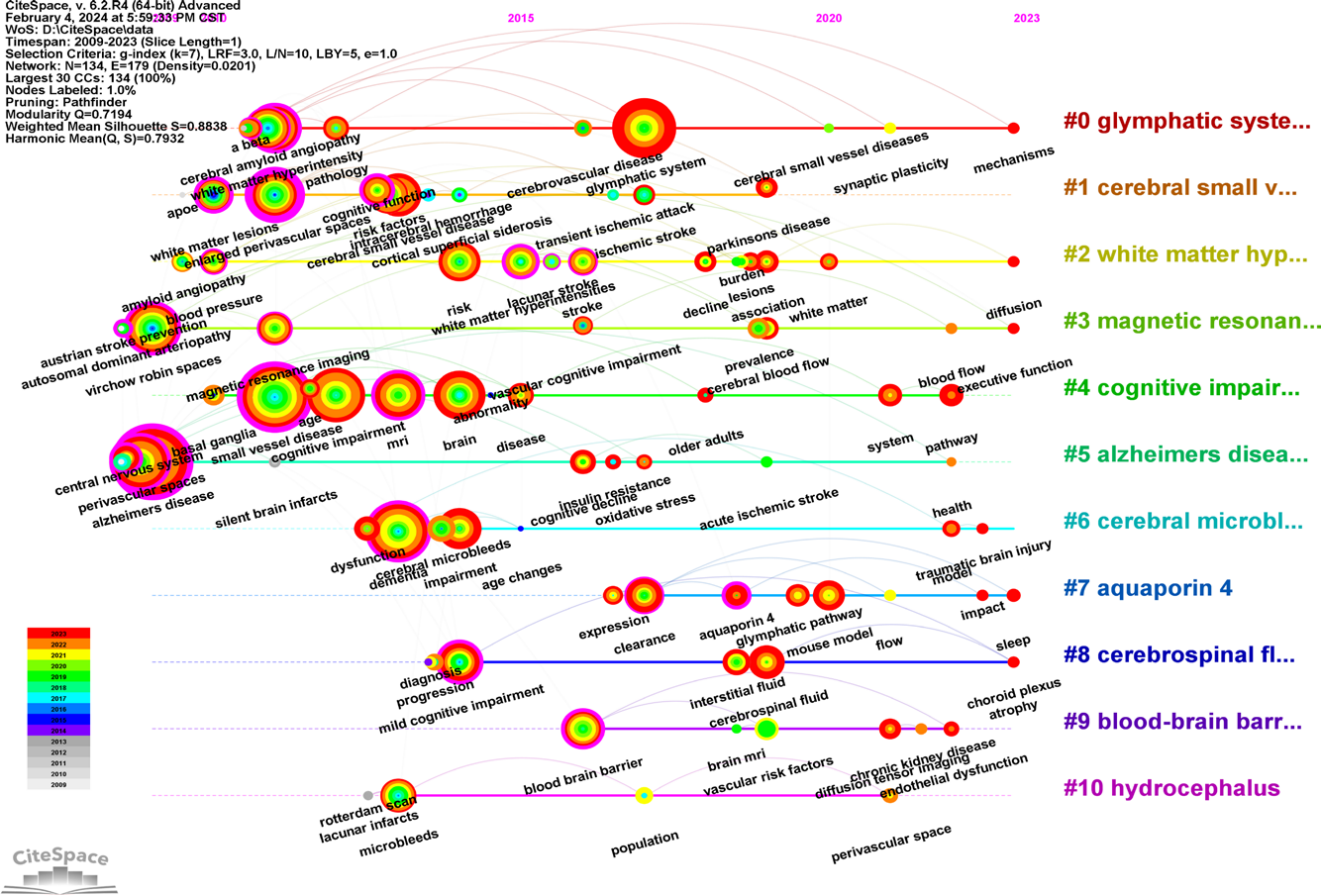
Keywords timeline analysis.

**Figure 12.**
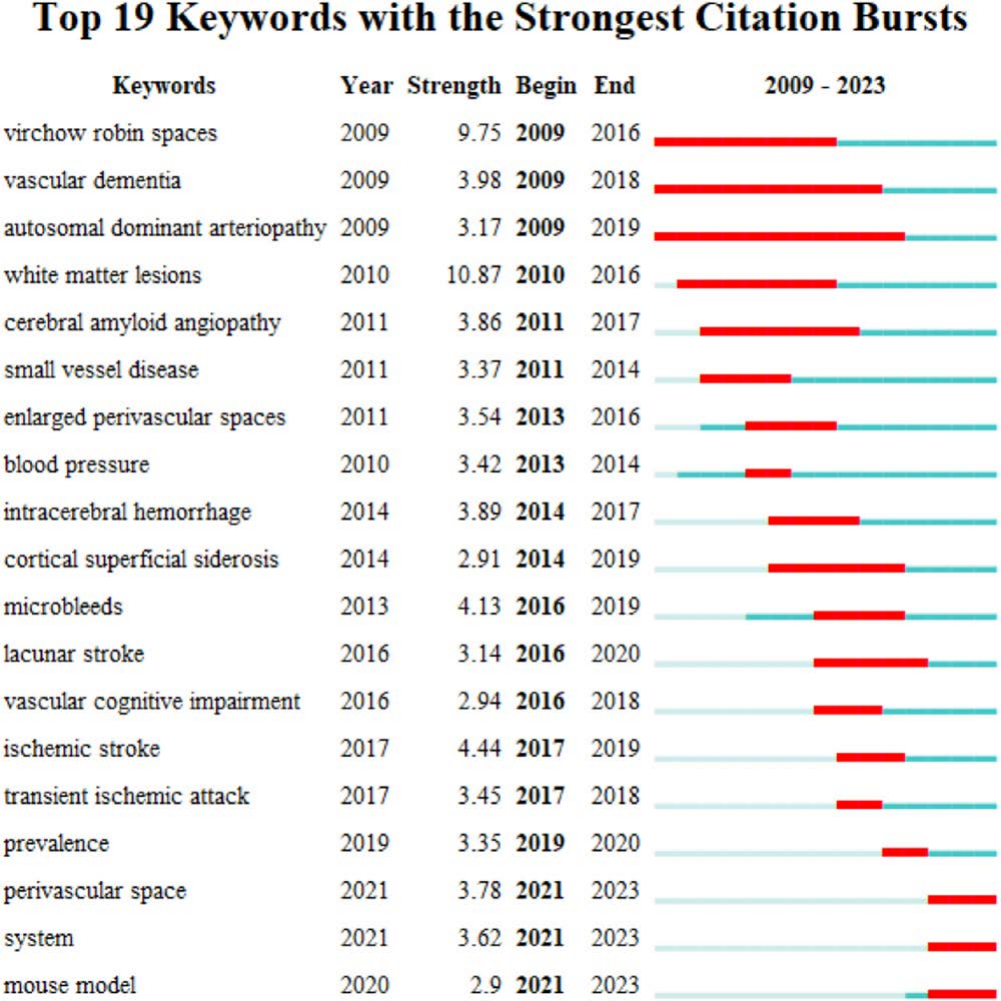
Keywords emergence analysis.

CiteSpace was used to present keywords timeline (Fig. 11) and emergence analyze (Fig. 12). AD and perivascular spaces had been a hot topic for a long time and from 2017 research on GS increased. Some studies on mechanisms and MRI in central nervous system were also hotspots. Research on a beta preceded research on AQP4 and clearance and this was also related to the time of GS concept. The keyword emergence analysis of the Web of Science Core Collection publications on GS in cognition generated 19 emergent terms. “Virchow robin spaces,” “vascular dementia,” and “autosomal dominant arteriopathy” were the first, and “white matter lesions” were the strongest. The longest-lasting keyword was white matter lesions. Studies on small vessel disease emerged from 2011 to 2014, and cerebral small vessel disease-related decreases in cognition were found to be associated with enlarged Virchow–Robin spaces.^[24]^ “System” and “mouse model” were words that have emerged in recent years.

## 4. Discussions

### 4.1. Key findings and insights from the literature

The bibliometric analysis revealed several influential studies that have shaped our understanding of the links between the GS and cognition. The most highly-cited article by Rasmussen et al^[5]^ provided a comprehensive overview of the glymphatic pathway’s role in neurological disorders, emphasizing its importance in Aβ clearance and the potential impact of impaired function on neurodegenerative diseases. This seminal review helped establish the GS as a critical player in cognitive health.

Subsequent high-impact studies have further elucidated the relationships between glymphatic function, AD pathology, and cognitive outcomes. Shokri-Kojori et al^[25]^ used PET imaging to demonstrate that a single night of sleep deprivation can lead to a significant increase in Aβ accumulation in the human brain, linking sleep disruption to AD risk via glymphatic clearance mechanisms. Kress et al^[26]^ showed that glymphatic transport and Aβ clearance decline with aging in mice, accompanied by reductions in CSF flow and AQP4 expression, providing a potential explanation for the increased incidence of AD and other dementias in older adults.

While these foundational studies have established the importance of the GS in cognitive function, there remain significant knowledge gaps and opportunities for future research. For example, the exact nature and extent of glymphatic dysfunction in different stages of AD and other neurodegenerative diseases is not yet fully characterized. The relative contributions of various factors like sleep, body position, and cardiovascular health to glymphatic function and cognitive outcomes also warrant further investigation.

Sleep,^[27]^ body posture,^[28]^ arterial pulsation,^[29]^ AQP4,^[3,30]^ and age^[26]^ have been identified as influential factors in glymphatic function and cognitive health. For instance, sleep has been shown to decrease fluid movement resistance, resulting in increased CSF–interstitial fluid exchange and more efficient solute clearance.^[31]^ Reduced sleep quality and duration have been associated with increased Aβ accumulation in the brain,^[25,32]^ which may contribute to cognitive impairment over time. Similarly, aging has been linked to declines in glymphatic transport and Aβ clearance, possibly due to reductions in CSF flow, arterial pulsatility, and AQP4 expression.^[26]^

Longitudinal studies in humans examining the relationships between imaging and fluid biomarkers of glymphatic clearance, neuroinflammation, and cognitive trajectories are needed to fully elucidate the role of this system in maintaining brain health across the lifespan. Multimodal imaging approaches, such as combining MRI, PET, and CSF biomarkers,^[16]^ may provide a more comprehensive understanding of the complex interactions between glymphatic function, neurodegeneration, and cognitive outcomes.

### 4.2. Challenges and future directions

Despite the growing body of evidence linking GS dysfunction to cognitive impairment and neurodegeneration, there remain significant challenges in this field. One major obstacle is the lack of standardized, noninvasive methods for assessing glymphatic function in humans. Current techniques like contrast-enhanced MRI and PET imaging have limitations in terms of safety, accessibility, and specificity. The development and validation of novel imaging approaches and fluid biomarkers for evaluating glymphatic clearance and waste accumulation in the human brain is a key priority for advancing research in this area.

Another challenge is the complex interplay between the GS and other factors influencing cognitive health, such as neuroinflammation, vascular dysfunction, and tau pathology. Disentangling the specific effects of impaired glymphatic clearance from these related processes will require carefully designed studies that integrate multiple imaging modalities, biomarker assessments, and cognitive outcome measures. Collaborative, interdisciplinary efforts will be essential for addressing these challenges and advancing our understanding of the GS’s role in maintaining brain health and preventing cognitive decline.

Promising directions for future research include the development of targeted therapies to enhance glymphatic clearance, such as pharmacological or noninvasive neuromodulation approaches. Recent studies have suggested that omega-3 polyunsaturated fatty acids,^[33]^ caffeoylquinic acid,^[34]^ and traditional Chinese medicine compounds like Xuefu Zhuyu decoction^[35]^ may promote Aβ clearance and improve cognitive function in animal models of AD and stroke. Additionally, acupuncture and related techniques, such as electroacupuncture and moxibustion, have shown potential for modulating glymphatic function and cognitive outcomes in animal studies.^[36,37]^ Further research is needed to determine the efficacy and mechanisms of these interventions in humans.

The identification of lifestyle factors and interventions that can optimize glymphatic function, such as sleep hygiene, physical activity, and dietary modifications, is also an important area for future investigation. Longitudinal studies examining the relationships between these modifiable risk factors, glymphatic clearance, and cognitive trajectories could provide valuable insights into potential prevention and treatment strategies.

In addition, exploring the potential of novel imaging techniques, such as diffusion tensor image analysis along the perivascular space (DTI-ALPS)^[38,39]^ and near-infrared spectroscopy,^[40]^ may provide new insights into the function and dysfunction of the GS in humans. Integrating these advanced imaging methods with established biomarkers and cognitive assessments could help elucidate the mechanisms underlying the relationship between glymphatic clearance and cognitive health across the lifespan.

Furthermore, investigating the role of the GS in other neurological disorders, such as Parkinson disease, multiple sclerosis, and traumatic brain injury, could expand our understanding of its importance in brain health and disease. Comparative studies across different patient populations and disease stages may help identify common mechanisms and potential therapeutic targets.

Ultimately, advancing our understanding of the GS and its role in brain health will require collaborative, interdisciplinary efforts that leverage cutting-edge technologies and innovative research strategies. By addressing the current challenges and pursuing promising avenues for future investigation, we may develop new approaches for the prevention and treatment of age-related cognitive decline and neurodegenerative diseases.

### 4.3. Limitations

First, due to the evolving terminology and lack of standardized nomenclature in this field, our search strategy may not have captured all relevant publications. While we attempted to include a broad range of keywords related to the GS and perivascular spaces, some studies may have been missed if they used alternative terms or descriptions.

Second, the bibliometric indicators used in this analysis, such as citation counts and keyword frequencies, provide a useful overview of research trends and impact but may not fully reflect the quality, originality, or significance of individual studies.

Third, as with any literature review, the findings and conclusions of this analysis are dependent on the accuracy, completeness, and timeliness of the underlying data sources. While the Web of Science Core Collection is a widely used and respected bibliographic database, it may not include all relevant publications, particularly those published in non-indexed journals.

## 5. Conclusions

In conclusion, this bibliometric analysis has revealed the rapid growth and evolving landscape of research on the GS and its role in cognitive function over the past 15 years. The increasing number of publications, particularly since the introduction of the GS concept in 2012, highlights the growing interest and importance of this topic. However, the high proportion of reviews compared to original research articles underscores the need for more innovative studies to fill current knowledge gaps and advance the field.

Key areas for future investigation include elucidating the mechanisms of glymphatic dysfunction in neurodegenerative diseases, developing standardized imaging and fluid biomarkers for assessing glymphatic function in humans, and identifying targeted interventions to enhance waste clearance and prevent cognitive decline. Addressing these challenges will require collaborative, interdisciplinary efforts and the application of cutting-edge technologies. By advancing our understanding of the critical role of the GS in maintaining brain health, we may ultimately develop new strategies for the prevention and treatment of age-related cognitive impairment and dementia.

## Author contributions

**Conceptualization:** Ying Zhang, Dexiong Han.

**Data curation:** Xiaoqi Ying.

**Formal analysis:** Xiaoqi Ying, Jingyang Xu.

**Funding acquisition:** Dexiong Han.

**Investigation:** Qintao Yu, Songsen Lan.

**Methodology:** Qintao Yu, Songsen Lan.

**Resources:** Jingyang Xu, Xinru Wang, Songsen Lan.

**Software:** Xinru Wang, Songsen Lan.

**Visualization:** Xiaoqi Ying, Jingyang Xu, Liwan Hu.

**Writing – original draft:** Xiaoqi Ying.

**Writing – review & editing:** Ying Zhang, Dexiong Han.
